# The relationship between reinforcement sensitivity and non-suicidal self-injury among adolescents: moderated mediation effect

**DOI:** 10.3389/fpsyg.2025.1603503

**Published:** 2025-11-07

**Authors:** Rong Kong, Ruihua Chen, Tingyu Hou, Na Li

**Affiliations:** 1 Center of Psychological Education and Counselling, Taiyuan Institute of Technology, Taiyuan, China; 2 School of Marxism, Beijing Polytechnic College, Beijing, China; 3 Hecheng Middle School, Pingyao, China; 4 Taiyuan No.13 Middle School, Taiyuan, China

**Keywords:** non-suicidal self-injury, reinforcement sensitivity, self-criticism, self-compassion, adolescents, psychological mediators

## Abstract

**Objective:**

Non-suicidal self-injury (hereinafter referred to as NSSI) is a significant public health concern among adolescents. Understanding its underlying psychological mechanisms is crucial for developing effective interventions. This study explored how reinforcement sensitivity affects NSSI among adolescents in China.

**Methods:**

A moderated mediation effect model was constructed to investigate the mediating role of self-criticism in the relationship between reinforcement sensitivity and NSSI, as well as the moderating role of self-compassion in this mediated pathway. A questionnaire survey was conducted with 1,582 middle school students. The study utilized established scales, including the BIS/BAS Scale, the Self-Criticism scale, the Self-Compassion Scale and the Adolescents Self-Harm Scale.

**Results:**

A total of 1,448 valid questionnaires were analyzed. The results showed that (1) punishment sensitivity can significantly positively predict NSSI among adolescents, with self-criticism partially mediating this relationship; (2) fun-seeking can significantly positively predict NSSI among adolescents, with self-criticism fully mediating this relationship; (3) reward responsiveness can significantly negatively predict NSSI among adolescents, with self-criticism partially mediating this relationship; (4) the relationship between self-criticism and NSSI was moderated by self-compassion (the latter half of the mediation effect).

**Conclusion:**

These results not only enhance our understanding of the mechanisms and conditions under which reinforcement sensitivity impacts adolescents’ NSSI, but also have important implications for targeted intervention measures.

## 1 Introduction

NSSI refers to the behavior that an individual intentionally and directly harms his own body without suicidal intention, and such behavior is not recognized by social norms (Nock, 2010; Sinclair et al., 2011; Yang et al., 2023). Common ways of NSSI include but are not limited to knife cuts, pokes, banging your head against a wall, electric shock, playing with fire in your hands, scalding, etc. (Feng, 2008; Nock, 2010). A recent meta-analysis of the incidence of NSSI among adolescents from a global sample showed that the overall average incidence of NSSI among adolescents over the past 5 years was 16%, with Asian adolescents having significantly higher rates than other continents (19.5 and 14.7%, respectively) (Farkas et al., 2024). A survey conducted in 2021 showed that the detection rate of NSSI among Chinese adolescents was 22.7% (Wu et al., 2021). NSSI is a significant risk factor for suicide and can significantly increase suicidal desire and risk (Klonsky et al., 2013). The 2019 Global Burden of Disease Data showed that self-injury was the third leading cause of disability-adjusted life years among adolescents aged 10–24, resulting in a significant economic burden (GBD 2019 Diseases and Injuries Collaborators, 2020). In view of the high incidence of NSSI among adolescents and the serious harm caused by NSSI, it is necessary to conduct in-depth research on the influencing factors and the factors’ mechanism.

Self-protection is an innate human instinct, representing individuals’ strongest, most inevitable, and most fundamental physiological and safety need. It manifests as automatic detection and defense against threatening stimuli, akin to a conditioned reflex. For example, when faced with pain, an individual’s limbs will quickly retract to avoid injury. A typical characteristic of NSSI, which is also difficult for normal individuals to understand, is that those who engage in NSSI do not dislike or avoid painful stimuli. Instead, they will unhesitatingly harm their own bodies in various ways. The benefits and barriers model of NSSI provides an explanation for this instinctive violation. This model proposes that NSSI carries many powerful benefits that are accessible to most people, such as emotional relief, self-punishment, punishment of others as well as seeking stimulation (Hooley and Franklin, 2018). However, if the “benefits” of NSSI are natural, widespread and powerful, why do many individuals who are experiencing emotional distress still refrain from NSSI? Is this due to differences in individuals’ responses to rewarding or punishing stimuli? The theory of reinforcement sensitivity points out that there are extensive individual differences in how sensitive individuals are to rewarding or punishing stimuli, and these differences are closely related to many psychological and mental disorders (Bijttebier et al., 2009). Reinforcement sensitivity refers to the tendency and degree of change in an individual’s behavioral and emotional responses when faced with stimuli, including punishment sensitivity and reward sensitivity (Ramer et al., 2024; Smillie et al., 2006; Ying et al., 2016). Punishment sensitivity reflects an individual’s reactivity to the appearance of punishment or the withdrawal of reward. In these two scenarios, individuals with high punishment sensitivity experience more negative emotions and exhibit more inhibitory and avoidant behaviors. Reward sensitivity reflects an individual’s reactivity to the appearance of reward or the withdrawal of punishment. In these two scenarios, individuals with high reward sensitivity experience more positive emotions and exhibit more approach behaviors (Bijttebier et al., 2009; Guo et al., 2009). In the case of individuals with NSSI, the mechanism behind the decision to use pathological reinforcers (i.e., NSSI) may be an anomaly in their reinforcement sensitivity.

Empirical studies have confirmed that reinforcement sensitivity abnormality is an important risk factor for the occurrence of NSSI (Jenkins et al., 2013; Liu and Liu, 2023; Liu, 2023; Robertson et al., 2013; Wu et al., 2021; Ying et al., 2016). A study of 136 men receiving treatment for drug addiction found that self-injurers showed a tendency to score higher for seeking novel stimuli than drug-dependent men who did not self-injure (Evren and Evren, 2006). Research on a non-clinical sample of college students revealed that increased reward sensitivity was associated with both the frequency and history of NSSI, but there was no significant correlation between punishment sensitivity and NSSI behavior. Some researchers questioned this. Their study indicated that both punishment sensitivity and reward sensitivity could effectively predict NSSI of college students (Liu, 2023; Ying et al., 2016), with punishment sensitivity even having a stronger prediction effect (Liu and Liu, 2023).

On the whole, although previous studies have confirmed the effect of reinforcement sensitivity on NSSI, the specific effect of punishment sensitivity and reward sensitivity on NSSI is not clear. Based on the existing theories and empirical evidence mentioned above, the present study hypothesized that both punishment sensitivity and reward sensitivity had positive predictive effects on NSSI. In addition, existing research lacks an in-depth exploration of the intrinsic mechanisms linking reinforcement sensitivity and NSSI, unable to answer questions such as how reinforcement sensitivity “works” on adolescents’ NSSI and under what conditions it “works.”

Self-criticism was first proposed by Blatt and Zuroff (1992). They argued that self-criticism was an individual’s ability to generate negative perceptions and harsh criticism about their own behavior, characterized by setting too strict standards for themselves, fearing disapproval or criticism from others, being extremely self-critical, pursuing perfectionism, and gaining recognition from important others through excessive compensation (Blatt and Zuroff, 1992). The defective self-model of NSSI posits that individuals with a high cognitive style of self-criticism are more likely to harm themselves because they believe they have flaws and deserve punishment (Hooley et al., 2010). Empirical studies have also confirmed the relationship between self-criticism and NSSI. A meta-analysis found a moderate to strong positive correlation between self-criticism and NSSI (Zelkowitz and Cole, 2019), with self-criticism significantly correlated with the frequency of NSSI (Gu et al., 2018). In a longitudinal study conducted in 2020, the level of self-criticism of eating disorder participants was significantly positively correlated with the number of self-injuries 2 months later (Perkins et al., 2020). It is worth noting that self-criticism is considered a cognitive style associated with reward sensitivity, which can predict a higher likelihood of bipolar disorder in individuals with high reward sensitivity (Stange et al., 2013). Some researchers have also verified that self-criticism played a mediating role between reward sensitivity and lifetime frequency of NSSI (Burke et al., 2015). Individuals with high reward sensitivity are more likely to be self-critical, which in turn increases their likelihood of engaging in NSSI. The defective self-model of NSSI also holds that individuals choose NSSI to satisfy the desire for self-punishment associated with self-critical cognitive style (Hooley et al., 2010). Based on the relationship between reinforcement sensitivity, self-criticism and NSSI, the present study hypothesized that self-criticism served as a mediating variable linking reinforcement sensitivity and NSSI.

Self-compassion is a concept derived from Buddhist psychology, first proposed by Neff (2003), which refers to an individual’s ability to express compassion and understanding towards themselves when facing difficulties or failures, emphasizing that treating one’s imperfections with kindness is more beneficial than self-criticism (Jin et al., 2020; Neff, 2003). Research showed that participants with a history of self-injury had significantly lower levels of self-compassion compared to those without such a history (Gregory et al., 2017). The participants were further divided into the current group of NSSI (had a history of NSSI last year), the past group of NSSI (had a history of NSSI 1 year ago) and the never-NSSI group. It was found that there were significant differences in the level of self-compassion among the three groups, with the current group having the lowest level of self-compassion and the never-group having the highest self-compassion (Gonçalves et al., 2024). According to the benefits and barriers model of NSSI, a positive self-view, which involves having a favorable opinion of oneself, will make individuals averse to NSSI, and a lack of compassion for themselves may help eliminate this barrier to NSSI, potentially leading individuals to believe that they should endure pain (Hooley et al., 2010; Hooley and Franklin, 2018).

Self-criticism often manifests as negative self-judgment and self-evaluation, so high self-critics are more likely to produce psychological pain and trigger self-injury behavior (Kirtley et al., 2015). Self-compassion, as an effective emotion regulation strategy, can significantly alleviate the negative effects of self-criticism on individuals (Doerig et al., 2014). High level self-compassion tends to alter how individuals interpret and evaluate situational events. On the one hand, self-compassion can help individuals reassess their perceptions of the importance of stressors and minimize their negative effects. On the other hand, self-compassion can help individuals identify possible positive consequences of the event, which in turn reduces responses to painful emotions, and the reduction of these painful emotions further decreases the risk of NSSI (Fritz, 2020). Considering the significant mitigating effect of self-compassion on NSSI, this study hypothesized that self-compassion can regulate the second stage of the proposed mediation pathway (from self-criticism to NSSI) among adolescents.

**FIGURE 1:**
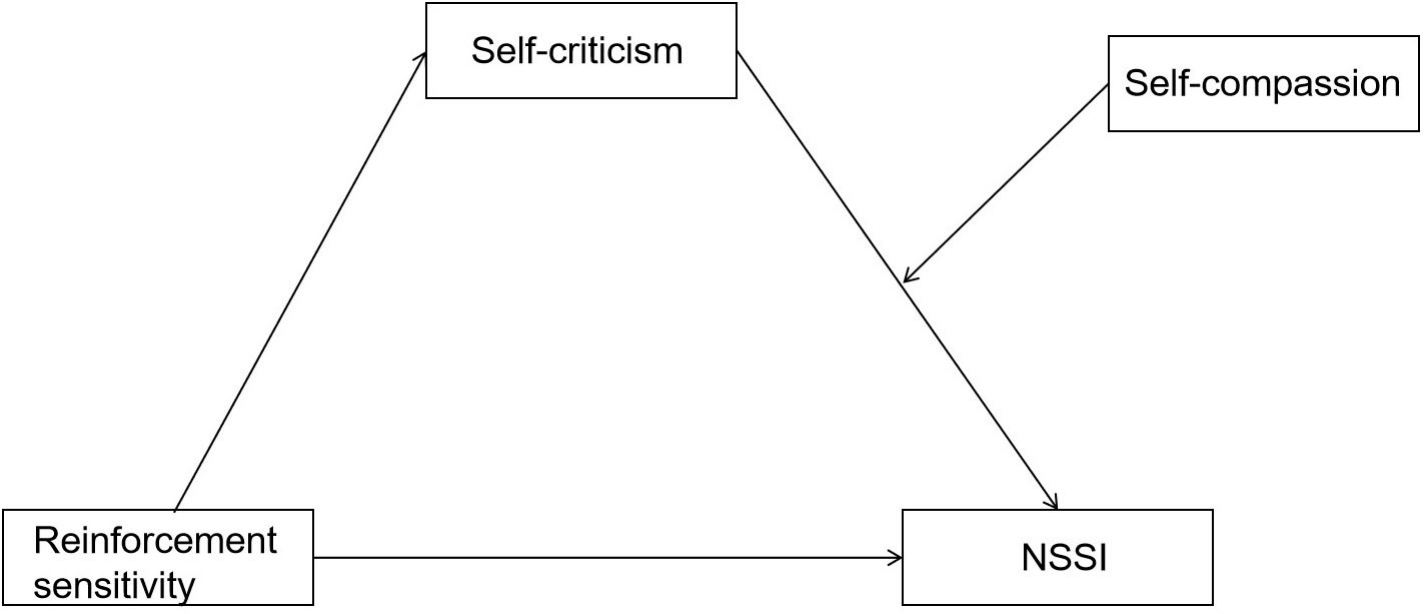
Hypothesized moderated mediation model.

In summary, the present study constructs a moderated mediation effect model (see Figure 1) to examine the relationships between reinforcement sensitivity, self-criticism, self-compassion, and NSSI. The specific hypotheses of this study are as follows:

H1: Both punishment sensitivity and reward sensitivity have positive predictive effects on NSSI.

H2: Self-criticism mediates the relationship between reinforcement sensitivity and NSSI.

H3: Self-compassion moderates the second stage of the mediating pathway (from self-criticism to NSSI), such that the effect of self-criticism on NSSI is weaker for individuals with higher levels of self-compassion.

## 2 Materials and methods

### 2.1 Participants

In a middle school in Taiyuan City and a middle school in Jinzhong City of Shanxi Province, a total of 1,582 questionnaires were distributed to high middle school students according to stratified cluster sampling method. The two participating schools were located in the urban area of North China. After removing 134 invalid questionnaires, 130 with a missing answer rate of more than 10% and 4 with extreme value, 1,448 valid questionnaires were collected, with an effective rate of 91.53%. There were 790 boy students, accounting for 54.6%, and 658 girl students, accounting for 45.4%. The youngest participant was 14 years old, the oldest was 19 years old, with an average age of 16.91 ± 1.21. One student did not provide age information. This study was conducted in accordance with the Declaration of Helsinki and was approved by the Ethics Committee of Beijing Vocational College of Industry Technology on September 23, 2023. Prior to data collection, the participating schools, the student participants and their parents were informed about the research’s objectives, confidentiality and anonymity; informed consent was obtained from all parties.

### 2.2 Measures

#### 2.2.1 NSSI

The Adolescents Self-Harm Scale (ASHS) (Feng, 2008) is a self-report questionnaire that assesses respondents’ self-injurious behavior in the absence of suicidal intent during the past year. This scale consists of 19 items, with Item 19 being an open-ended question (if you had any other intentional methods of self-injury not mentioned in this questionnaire, please write them down). The other 18 items are divided into two parallel sections: frequency of certain forms of NSSI and degree of physical injury caused by NSSI.

The frequency section is scored at 4 points (0 = never, 1 = once, 2 = twice to four times, 3 = five times or more). The degree section is scored at five points (0 = none, 1 = mild, 2 = moderate, 3 = severe, 4 = extremely severe). The total score of the questionnaire is the sum of the products of all items in both sections, with higher scores indicating more severe self-injurious behavior. In the original study (Feng, 2008), adequate internal consistency was found (α = 0.85). In the present study adequate internal consistency was also obtained (α = 0.94).

#### 2.2.2 Reinforcement sensitivity

The Chinese version of the BIS/BAS Scale (Carver and White, 1994) was used to measure participants’ reinforcement sensitivity (Li et al., 2008). This scale consists of 18 items and is comprised of one BIS subscale and three BAS subscales (i.e. BAS Drive, BAS Fun-Seeking, and BAS Reward-Responsiveness). The BIS subscale measures punishment sensitivity, while the other three subscales measure reward sensitivity: BAS Drive reflects the degree to which desired goals are pursued; BAS Fun-Seeking reflects the degree to which rewarding and novel experiences are desired and approached; BAS Reward-Responsiveness reflects the degree to which participants experience positive affect in the context of potential reward.

Responses are made on a 4-point Likert scale ranging from 1 (very false for me) to 4 (very true for me), with higher scores indicating higher level of reinforcement sensitivity. In previous studies on reinforcement sensitivity, this scale has shown acceptable psychometric properties (Wu et al., 2021). In this study, the Cronbach’s alpha coefficient was 0.854 for BIS, 0.834 for BAS Drive, 0.779 for BAS Fun-seeking, and 0.824 for BAS Reward-Responsiveness.

#### 2.2.3 Self-criticism

The Chinese version of the self-criticism subscale from the Depression Experience Questionnaire (Bagby et al., 1994) was used to measure participants’ self-criticism. This subscale consists of eight items such as “I have a difficult time accepting weaknesses in myself.” Responses are made on a 5-point scale ranging from 0 (does not apply to me at all) to 5 (applies to me completely), with higher scores indicating higher level of self-criticism. In previous studies on self-criticism, this scale has shown good psychometric properties (Burke et al., 2015). In this study, the Cronbach’s α coefficient for this scale is 0.840.

#### 2.2.4 Self-compassion

The Chinese version of the Self-Compassion Scale (Neff, 2003), a 12-item self-report measure, was used to measure participants’ self-compassion. Responses are made on a 5-point scale ranging from 1 (almost never) to 5 (almost always), with higher scores indicating higher level of self-compassion. The Chinese version has shown good psychometric properties in previous studies (Gong et al., 2014). In this study, the Cronbach’s α coefficient for the scale is 0.804.

### 2.3 Data analysis

The data was edited, organized, coded and entered in SPSS 23.0. We first used SPSS 23.0 to compute descriptive statistics for all variables and the bivariate correlations between them. Next, we tested the hypothesized mediation model using Model 4 of PROCESS (Hayes and Scharkow, 2013). Model 14 was used to analyze whether the mediating effect (the latter part) was moderated by self-compassion (Hayes and Scharkow, 2013).

## 3 Results

### 3.1 Common method bias

To address the potential for common method bias (a systematic error that threatens validity by introducing spurious correlations among variables measured with the same method) inherent in self-report data, both procedural and statistical remedies were employed. The results indicated that common method bias was not a serious concern in this study. Procedurally, we minimized this risk by using different response scales (e.g., agreement vs. frequency) across questionnaires. Statistically, Harman’s single-factor test was conducted (Zhou and Long, 2004). The analysis revealed 13 factors with eigenvalues greater than 1, with the first factor accounting for only 19.43% of the variance, which is well below the critical threshold of 40%.

### 3.2 Descriptive statistics and correlations

Table 1 lists the mean, standard deviation and Pearson correlation matrices of each variable. The results showed that there were significant positive correlations between punishment sensitivity, fun-seeking, self-criticism and NSSI. Reward responsiveness was negatively correlated with NSSI and positively correlated with self-criticism. There was no significant correlation between drive and NSSI, but a significant positive correlation between drive and self-criticism. Self-compassion was negatively correlated with NSSI, self-criticism, punishment sensitivity and fun seeking, while positively correlated with reward responsiveness. Reward sensitivity was positively correlated with self-criticism, but not correlated with self-compassion and NSSI. There was a significant positive correlation between the four dimensions of reinforcement sensitivity.

**TABLE 1:** Mean, standard deviation and correlation matrix of each variable (**n** = 1,448).

Variable	1	2	3	4	5	6	7	8
1 NSSI	1	0.19^**^	−0.22^**^	0.11^**^	−0.03	0.05*	−0.06*	−0.01
2 Self-criticism		1	−0.59^**^	0.54^**^	0.07*	0.28^**^	0.11^**^	0.20^**^
3 Self-compassion			1	−0.41^**^	0.14^**^	−0.16^**^	0.09^**^	0.01
4 Punishment sensitivity				1	0.20^**^	0.36^**^	0.41^**^	0.39^**^
5 Drive					1	0.48^**^	0.57^**^	0.82^**^
6 Fun-seeking						1	0.48^**^	0.82^**^
7 Reward responsiveness							1	0.81^**^
8 Reward sensitivity								1
M ± SD	3.28 ± 9.69	24.84 ± 6.97	39.41 ± 7.78	14.45 ± 3.22	11.47 ± 2.49	14.02 ± 2.92	12.72 ± 2.31	38.21 ± 6.33

*p < 0.05,

^**^p < 0.01, NSSI, non-suicidal self-injury.

### 3.3 Regression analyses with reinforcement sensitivity predicting NSSI

The regression analyses revealed that both punishment sensitivity and reward sensitivity significantly predicted NSSI, though in opposite directions, even after controlling for the effect of gender. Furthermore, when examining the subdimensions of reward sensitivity, reward responsiveness and fun-seeking emerged as significant predictors of NSSI, whereas drive did not demonstrate a significant effect. These results were derived from two hierarchical regression models: Model 1 included punishment sensitivity and overall reward sensitivity as predictors, while Model 2 further decomposed reward sensitivity into its three subdimensions. Detailed results are presented in Table 2.

**TABLE 2:** Regression analysis with reinforcement sensitivity predicting NSSI.

Regression model	Independent variable	β	SE	*t*	*p*	*R* ^2^	*F*	Δ *R*^2^
Model 1	Punishment sensitivity	0.14	0.09	4.97^***^	0.000	0.02	8.68^***^	0.02
	Reward sensitivity	−0.06	0.04	−2.25*	0.024			
Model 2	Punishment sensitivity	0.15	0.09	5.25^***^	0.000	0.03	9.70^***^	0.03
	Drive	−0.01	0.13	−0.26	0.797			
	Fun-seeking	−0.08	0.11	2.45*	0.014			
	Reward responsiveness	−0.16	0.14	−4.53^***^	0.000			

*p < 0.05,

^***^p < 0.001, NSSI, non-suicidal self-injury.

### 3.4 Test of the mediating role of self-criticism

On the basis of correlation analysis, with punishment sensitivity, fun-seeking, and reward responsiveness as independent variables, NSSI as the dependent variable, and self-criticism as the mediating variable, bias-corrected non-parametric percentile Bootstrap was used to test the mediation effect under the premise of controlling gender. A random sample of 5,000 samples was drawn from the original data to calculate the 95% confidence interval. If the 95% confidence interval for both the total effect path and the mediation effect path do not include 0, it indicates that the mediating role of self-criticism is valid.

The mediation analysis revealed that self-criticism played a significant mediating role between each dimension of reinforcement sensitivity and NSSI, though the nature of mediation differed across predictors. Specifically, self-criticism partially mediated the relationship between punishment sensitivity and NSSI, as well as between reward responsiveness and NSSI, while it fully mediated the effect of fun-seeking on NSSI.

First, with punishment sensitivity as the independent variable, self-criticism as the mediating variable and NSSI as the dependent variable. Results showed that after adding self-criticism, punishment sensitivity could significantly positively predict NSSI (β = 0.212, *p* < 0.05), self-criticism could also significantly positively predict NSSI (β = 0.209, *p* < 0.001). The results of mediation analysis showed that the direct effect corresponding to punishment sensitivity was significant(effect value was 0.212, SE = 0.102, 95% confidence interval was [0.012, 0.412] and the indirect effect of self-criticism was significant (mediation effect value was 0.255, SE = 0.052, 95% confidence interval was [0.155, 0.358]), indicating that self-criticism had a partial mediating effect between punishment sensitivity and NSSI, with the mediating effect accounting for 54.7% of the total effect.

Second, with fun-seeking as the independent variable, self-criticism as the mediating variable and NSSI as the dependent variable. Results showed that after adding self-criticism, fun-seeking was no longer a significant predictor of NSSI (β = 0.162, *p* > 0.05), self-criticism could significantly positively predict NSSI (β = 0.209, *p* < 0.001). The results of mediation analysis showed that the direct effect corresponding to fun-seeking was not significant (effect value was 0.162, SE = 0.101, 95% confidence interval was [-0.035, 0.360] and the indirect effect of self-criticism was significant (mediation effect value was 0.086, SE = 0.021, 95% confidence interval was [0.048, 0.131]), indicating that self-criticism had a complete mediating effect between fun-seeking and NSSI, with the mediating effect accounting for 34.5% of the total effect.

Third, with reward responsiveness as the independent variable, self-criticism as the mediating variable and NSSI as the dependent variable. Results showed that after adding self-criticism, reward responsiveness could significantly positively predict NSSI (β = -0.543, *p* < 0.001), self-criticism could significantly positively predict NSSI (β = 0.209, *p* < 0.001). The results of mediation analysis showed that the direct effect corresponding to reward responsiveness was significant (effect value was -0.543, SE = 0.131, 95% confidence interval was [-0.799, -0.286] and the indirect effect of self-criticism was significant (mediation effect value was -0.127, SE = 0.029, 95% confidence interval was [-0.189, -0.074]), indicating that self-criticism had a partial mediating effect between reward responsiveness and NSSI, with the mediating effect accounting for 19.0% of the total effect.

### 3.5 Test of the moderating effect of self-compassion on the mediating effect of self-criticism

We next tested whether self-compassion moderates the second stage of the mediation pathway (from self-criticism to NSSI). This was examined for each of the three independent variables (punishment sensitivity, fun-seeking, reward responsiveness) using the PROCESS macro for SPSS (Hayes and Scharkow, 2013).

A consistent and statistically significant pattern emerged across all three models (see Tables 3–5). In each case, the interaction term between self-criticism and self-compassion was a significant negative predictor of NSSI (e.g., for punishment sensitivity: β = -0.020, *p* < 0.001).

**TABLE 3:** The moderating effect of self-compassion (punishment sensitivity as the independent variable).

Regression equation		Overall fit index			Significance of regression coefficients		
Dependent variable	Independent variable	*R*	*R* ^2^	*F*	β	*t*	95%CI
NSSI	Gender				−0.814	−1.632	[−1.791, 0.164]
	Self-criticism				0.135	2.833^**^	[0.042, 0.229]
	Self-compassion				−0.215	−5.426^***^	[−0.292, −0.137]
	Self-compassion × self-criticism	0.268	0.072	22.319^***^	−0.020	−5.105^***^	[−0.027, −0.012]

^**^p < 0.01,

^***^p < 0.001.

**TABLE 4:** The moderating effect of self-compassion (fun seeking as the independent variable).

Regression equation		Overall fit index			Significance of regression coefficients		
Dependent variable	Independent variable	*R*	*R* ^2^	*F*	β	*t*	95%CI
NSSI	Gender				−0.824	−1.667	[−1.794, 0.146]
	Self-criticism				0.135	2.990*	[0.046, 0.223]
	Self-compassion				−0.214	−5.453^***^	[−0.291, −0.137]
	Self-compassion × self-criticism	0.268	0.072	22.327^***^	−0.020	−5.108^***^	[−0.028, −0.012]

*p < 0.05,

^***^p < 0.001.

**TABLE 5:** The moderating effect of self-compassion (reward responsiveness as the independent variable).

Regression equation		Overall fit index			Significance of regression coefficients		
Dependent variable	Independent variable	*R*	*R* ^2^	*F*	β	*t*	95%CI
NSSI	Gender				−0.768	−1.556	[−1.736, 0.200]
	Self-criticism				0.160	3.578^***^	[0.072, 0.247]
	Self-compassion				−0.191	−4.788^***^	[−0.269, −0.113]
	Self-compassion × self-criticism	0.278	0.077	24.192^***^	−0.021	−5.473^***^	[−0.029, −0.014]

^***^p < 0.001.

**FIGURE 2:**
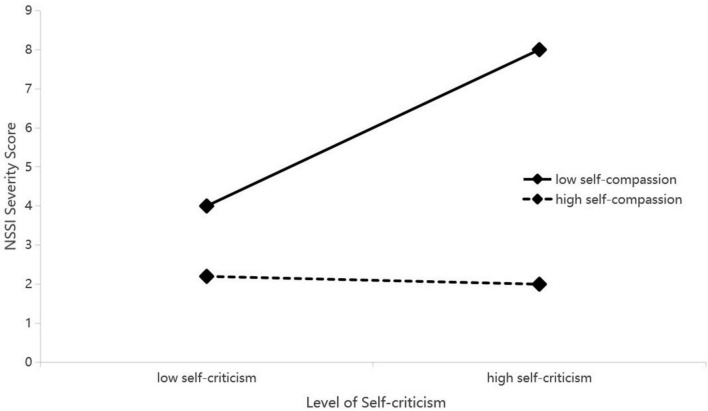
Interaction of self-criticism by self-compassion predicting NSSI. Low and high values represent –1 and +1 standard deviation, respectively. See Table 3 for interaction statistics.

**FIGURE 3:**
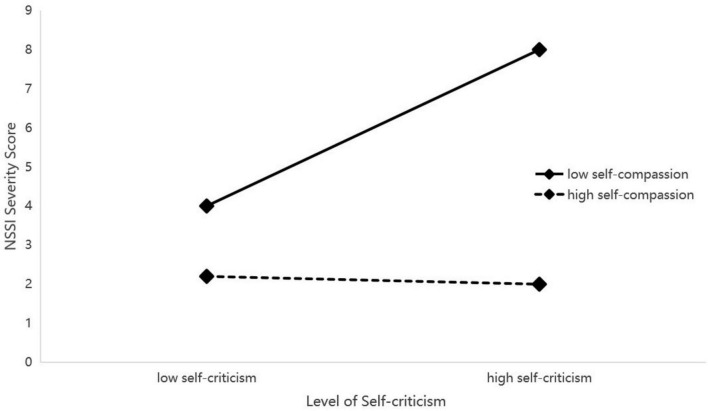
Interaction of self-criticism by self-compassion predicting NSSI. Low and high values represent –1 and +1 standard deviation, respectively. See Table 4 for interaction statistics.

**FIGURE 4:**
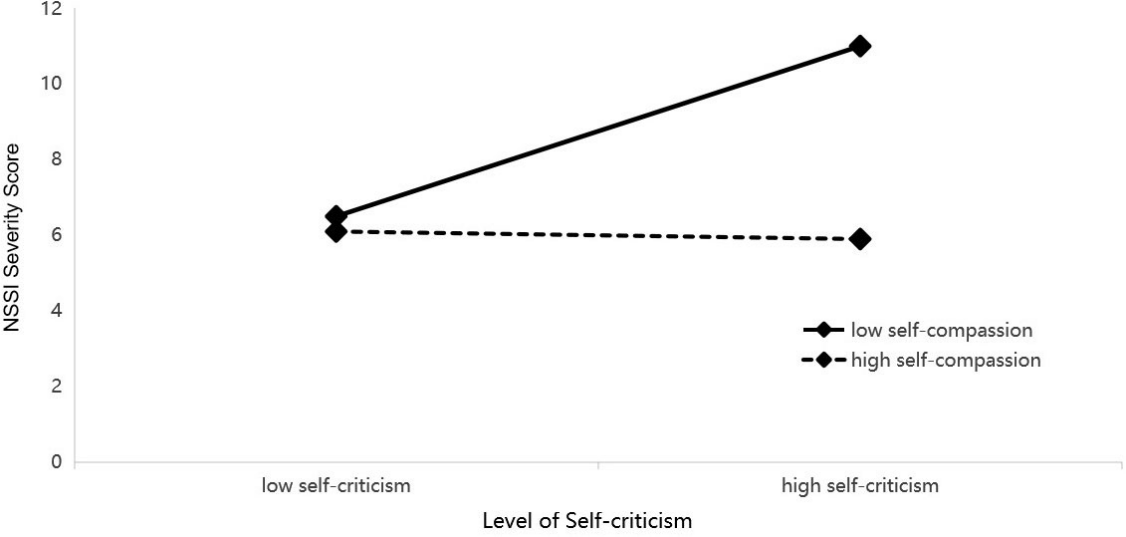
Interaction of self-criticism by self-compassion predicting NSSI. Low and high values represent -1 and +1 standard deviation, respectively. See Table 5 for interaction statistics.

To interpret this interaction, simple slope analyses were performed (see Figures 2–4). The analyses uniformly showed that for adolescents with low levels of self-compassion, self-criticism was a strong and significant positive predictor of NSSI (e.g., *b*_*simple*_ = 0.289, *p* < 0.001). Conversely, for those with high levels of self-compassion, the relationship between self-criticism and NSSI was not significant (e.g., *b*_*simple*_ = −0.019, *p* > 0.05).

Conditional indirect effect analyses confirmed the implications of this moderation. The indirect effect of each reinforcement sensitivity dimension on NSSI through self-criticism was significant only at low levels of self-compassion (e.g., for punishment sensitivity: effect = 0.340, 95% CI [0.156, 0.523]). At high levels of self-compassion, this indirect pathway was effectively neutralized. The index of moderated mediation was significant for all three models, confirming that self-compassion significantly buffers the mediated pathway from reinforcement sensitivity to NSSI via self-criticism.

## 4 Discussion

Adolescents have a high incidence of NSSI, and in recent years, the rate has been on the rise, making it an important public health issue that threatens the physical and mental development of adolescents (Hudson et al., 2024; Sinclair et al., 2011). This study focused on adolescents and examined the impact of reinforcement sensitivity, self-criticism, and self-compassion on NSSI through a moderated mediation model. The findings indicated that punishment sensitivity and fun-seeking were risk factors for NSSI, while reward responsiveness was a protective factor. Meanwhile, self-criticism played a partial mediating role in the relationship between punishment sensitivity and NSSI, as well as between reward responsiveness and NSSI. It also played a complete mediating role in the relationship between fun-seeking and NSSI. The mediation role was moderated by self-compassion. The results of this study are helpful to understand the mechanism of NSSI among high school students and provide suggestions for preventing NSSI.

### 4.1 The effect of reinforcement sensitivity on predicting NSSI

In this study, the effect size of the regression equation with punishment sensitivity and the three dimensions of reward sensitivity as independent variables was higher than that of the regression equation with punishment sensitivity and the total score of reward sensitivity as independent variables. This may be because the three dimensions of reward sensitivity focused on different aspects and had different effects on NSSI behavior and the drive dimension was not significantly related to NSSI. Therefore, using the total score of reward sensitivity as an independent variable lost some information, affecting the explanatory power of the equation to some extent.

Punishment sensitivity had a positive predictive effect on NSSI. The higher the punishment sensitivity, the higher the level of NSSI. This finding is consistent with previous research (Liu, 2023; Wu et al., 2021; Ying et al., 2016) and can be explained by the connection between punishment sensitivity, emotion regulation disorder and NSSI. Individuals with high sensitivity to punishment are more responsive to punishment stimuli, making them more prone to intense negative emotions when faced with negative or stressful situations (Bijttebier et al., 2009). This directly or indirectly leads to difficulty in emotional regulation, preventing individuals from effectively coping with (Dillien et al., 2023; Tull et al., 2010). In such intense negative emotions, individuals may resort to NSSI to detach themselves from aversive and negative experiences, thereby achieving emotional regulation and reducing psychological distress (Yu, 2013). In this process, excessively high punishment sensitivity undoubtedly provides a foundation for the occurrence of this negative reinforcement effect.

Fun-seeking had a positive predictive effect on NSSI. The higher the pleasure-seeking, the higher the level of NSSI. This can be explained by the relationship between the function of NSSI and physiological benefits. For individuals with a high level of fun-seeking, boredom, emptiness, and nonexistent experiences are difficult to tolerate (Bijttebier et al., 2009). Existing studies have shown that NSSI leads to the release of endogenous opioid peptides, which may bring some form of pleasure (Bresin and Gordon, 2013; Kao et al., 2024). In this case, the physiological satisfaction brought by NSSI is a rewarding experience, making such individuals more likely to engage in NSSI for the sake of seeking stimulation or pleasure. This confirms the results of previous studies on the function of NSSI, that is, one important function of NSSI is sensation seeking, which exists and is maintained in the form of intrapersonal positive reinforcement (Guérin-Marion et al., 2018; Knorr et al., 2013).

Reward responsiveness had a negative predictive effect on NSSI. The higher the reward responsiveness, the lower the level of NSSI. This finding is inconsistent with the research hypothesis of this study and also contradicts previous studies. In Ying et al. (2016) study, reward responsiveness had no explanatory power on NSSI, whereas in this study, reward responsiveness had a protective effect on NSSI, with the effect even greater than the effect of fun-seeking on NSSI, showing strong statistical significance. The results of this study can be explained by evidence from previous studies. McLaughlin and Lambert (2017) found that high response to reward stimuli was a protective factor against increasing psychopathological risk in individuals with childhood trauma, while a sluggish pattern of positive reward response increased the vulnerability of such individuals to NSSI (Kautz et al., 2020). The possible reason is that a high response to reward makes it easier for individuals to experience positive emotions from their environment. Besides, according to the broaden-and-build theory of positive emotion, positive emotional experience can “correct” and “eliminate” individuals’ depression and other negative emotions, thus preventing self-harm (Burke et al., 2018).

The drive dimension had no explanatory power for NSSI. Drive is characterized by the relentless pursuit of goals (Carver and White, 1994), and Tull et al. (2010) also found that drive was negatively correlated with difficulty regulating emotions. It is evident that this dimension can hardly provide a basis for the occurrence of NSSI.

### 4.2 The mediating effect of self-criticism on the relationship between reinforcement sensitivity and NSSI

The results of this study support the hypothesis that self-criticism plays a mediating role between reinforcement sensitivity and NSSI among adolescents: high punishment sensitivity, high fun-seeking and high reward responsiveness lead to the development of a self-critical cognitive style, causing cognitive dissonance, which increases susceptibility to both positive and negative reinforcements for NSSI. In other words, self-criticism is one of the key mechanisms of reinforcement sensitivity affecting NSSI in high school students. This finding provides a possible explanation for the relationship between reinforcement sensitivity and NSSI from a cognitive perspective. The results are also consistent with previous findings. A study of 177 adolescents with high and moderate levels of reward sensitivity found that self-criticism mediated the relationship between reward sensitivity and the frequency of NSSI in the past year (Burke et al., 2015). In addition, self-criticism also mediated the relationship between reward sensitivity and lifetime frequency of NSSI (Burke et al., 2015). The previous findings indicated that cognitive regulation styles may help explain why individuals with high reward sensitivity were more likely to engage in NSSI, but the study did not explore the mechanisms underlying the relationship between punishment sensitivity and self-injury. NSSI is a non-adaptive coping way for adolescents to deal with emotional problems, which is partly due to their inappropriate cognitive style, namely, excessive self-criticism. Our results further highlight the strength of the relationship between self-criticism and self-injury.

### 4.3 The moderating effect of self-compassion on the mediating effect of self-criticism

This study found that self-compassion could significantly moderate the mediating effect of self-criticism on the relationship between reinforcement sensitivity and NSSI. Specifically, high levels of self-compassion attenuated the positive impact of self-criticism on NSSI, thereby weakening the indirect effect between reinforcement sensitivity and NSSI. For adolescents with higher levels of self-compassion, the influence of self-criticism on NSSI was no longer significant. The environmental function model of NSSI posits that cognitive beliefs are a crucial factor affecting NSSI. Self-criticism is a kind of negative self-judgment and self-evaluation that can significantly affect an individual’s mental health. Recently, self-compassion has been proposed as an important tool for improving mood and boosting happiness, and it has been proven to be a quality that can be cultivated through training (Germer and Neff, 2013; Neff and Germer, 2013). Neff (2003) described it as an attitude of positive self-perception and acceptance of one’s emotional experiences. People who are rich in self-compassion accept both pain and loss, allowing themselves to evaluate themselves with a non-judgmental attitude based on warmth and kindness, effectively countering the adverse effects of self-criticism (Neff, 2003). Recent brain imaging studies have shown that self-criticism can activate the right prefrontal cortex, while high levels of self-compassion can significantly reduce brain activity in this region (Doerig et al., 2014)

### 4.4 Implications for the prevention and intervention of NSSI among adolescents

Intervention programs based on reinforcement sensitivity theory have been successfully applied to various psychological disorders (Bijttebier et al., 2009). Our study provides empirical support for applying the RST framework to NSSI intervention. Clinicians can assess individual reinforcement sensitivity patterns to design different intervention programs for self-injurers. For adolescents with high punishment sensitivity, components of Dialectical Behavior Therapy, such as emotion regulation and distress tolerance skills (Amestoy et al., 2025), may be particularly effective in reducing avoidance-driven NSSI. For those with high reward sensitivity, Cognitive-Behavioral Therapy techniques like behavioral activation and managing reward contingencies (Cuijpers et al., 2023) could help channel reward-seeking into adaptive behaviors, reducing the risk of NSSI as a maladaptive coping strategy.

At the preventative level, parents can help mitigate the development of excessive punishment sensitivity by fostering a nurturing, low-critical environment and avoiding conditionally valued relationships. Similarly, encouraging a balanced attitude toward rewardsnced attitude voiding conditionally valued relationships. Similarly, encnishment sensitivity by fostering aeward sensitivity.

Furthermore, reducing self-criticism is crucial. Educators and clinicians should guide adolescents to develop positive attribution styles and self-regulation abilities. For students exhibiting self-critical tendencies, which often manifest as interpersonal hostility, providing adequate social support and psychological counseling is essential.

Finally, our finding that self-compassion buffers the link between self-criticism and NSSI provides empirical support for the mechanisms targeted in Compassion-Focused Therapy (CFT). CFT is specifically designed to reduce self-criticism and shame and enhance self-soothing abilities (Ferrari et al., 2019), making it a highly relevant approach for those with a high level of self-criticism. Therefore, integrating CFT techniques—such as compassionate mind training or compassionate imagery—could be particularly beneficial for self-injurers with high levels of self-criticism as identified in our study. Techniques from Mindfulness-Based Cognitive Therapy (MBCT), which redirect attention from rumination to present-moment experience, have also proven effective in increasing distress tolerance and reducing NSSI behaviors (Reese et al., 2015).

In school settings, evidence-based prevention programs that incorporate mindfulness and self-compassion training, such as “Learning to Breathe” can be implemented to improve emotional awareness and self-kindness among adolescents (Lucas-Thompson et al., 2020).

### 4.5 Limitations and future directions

There are some limitations which need to be further improved in future studies. First, all data in this study were collected using self-report measures completed by adolescents. Since NSSI is a stigmatized behavior often subject to social disapproval (Liu, 2023; Nock, 2010), self-reports may be affected by social desirability bias, potentially compromising the reliability of the findings. To improve validity in future research, it is recommended to incorporate multi-method assessment strategiesuture as behavioral tasks, clinical interviews, and multi-informant reports from parents, teachers, and peers. Second, it is important to note that due to the cross-sectional nature of our study, the observed relationships represent associations and cannot be interpreted as evidence of causality. Therefore, future research should adopt longitudinal designs or clinical intervention studies to clarify the temporal order and causal relationships between these variables. Third, in terms of the mediating mechanisms, the present study showed that self-criticism only partially mediates the relationship between punishment sensitivity and NSSI, as well as reward responsiveness and NSSI, suggesting that there may be other mediating variables. This study examined the cognitive mediating variable of self-criticism, and future research should consider the emotional or physiological mediating variables.

## 5 Conclusion

The present study constructed a moderated mediation model to elucidate the mechanisms linking reinforcement sensitivity to adolescent NSSI. The findings both confirmed and refined our initial hypotheses.

Contrary to H1, which posited a positive prediction from all reinforcement sensitivity dimensions, the results revealed a more nuanced picture: punishment sensitivity and the fun-seeking component of reward sensitivity were positive predictors of NSSI, whereas reward responsiveness was a significant negative predictor.

In support of H2, self-criticism was identified as a key mediator in these relationships. It partially mediated the effects of both punishment sensitivity and (the negatively correlated) reward responsiveness on NSSI, and fully mediated the effect of fun-seeking.

Finally, H3 was strongly supported. Self-compassion significantly moderated the pathway from self-criticism to NSSI. Specifically, the impact of self-criticism on NSSI was mitigated among adolescents with higher levels of self-compassion, highlighting its potent protective role.

## Data Availability

The raw data supporting the conclusions of this article will be made available by the authors, without undue reservation.
